# Genome-wide identification and localization of chalcone synthase family in soybean (*Glycine max* [L]Merr)

**DOI:** 10.1186/s12870-018-1569-x

**Published:** 2018-12-04

**Authors:** Arun Kumaran Anguraj Vadivel, Kevin Krysiak, Gang Tian, Sangeeta Dhaubhadel

**Affiliations:** 1 London Research and Development Centre, Agriculture and Agri-Food Canada, 1391 Sandford Street, London, Ontario N5V 4T3 Canada; 2 Department of Biology, University of Western Ontario, London, ON Canada

**Keywords:** Chalcone synthase, Isoflavonoid, Flavonoid, Gene duplication, Gene expression, Soybean, Gene family

## Abstract

**Background:**

Soybean is a paleopolyploid that has undergone two whole genome duplication events. Gene duplication is a type of genomic change that can lead to novel functions of pre-existing genes. Chalcone synthase (CHS) is the plant-specific type III polyketide synthase that catalyzes the first committed step in (iso)flavonoid biosynthesis in plants.

**Results:**

Here we performed a genome-wide search of *CHS* genes in soybean, and identified 21 GmCHS loci containing 14 unique *GmCHS* (*GmCHS1-GmCHS14*) that included 5 newly identified *GmCHS*s (*GmCHS10-GmCHS14*). Furthermore, 3 copies of *GmCHS3* and 2 copies of *GmCHS4* were found in soybean. Analysis of gene structure of *GmCHS*s revealed the presence of a single intron in protein-coding regions except for *GmCHS12* that contained 3 introns. Even though *GmCHS* genes are located on 8 different chromosomes, a large number of these genes are present on chromosome 8 where they form 3 distinct clusters. Expression analysis of *GmCHS* genes revealed tissue-specific expression pattern, and that some GmCHS isoforms localize in the cytoplasm and the nucleus while other isoforms are restricted to cytoplasm only.

**Conclusion:**

Overall, we have identified 21 GmCHS loci with 14 unique *GmCHS* genes in the soybean genome. Their gene structures and genomic organization together with the spatio-temporal expression and protein localization suggest their importance in the production of downstream metabolites such as (iso)flavonoids and their derived phytoalexins.

**Electronic supplementary material:**

The online version of this article (10.1186/s12870-018-1569-x) contains supplementary material, which is available to authorized users.

## Background

Whole genome duplication has occurred multiple times over the past 200 millions of years of plant evolution leading to gene duplications. The availability of whole genome sequences of a large number of plant species has shown that approximately 64.5% of plant genes are duplicated (reviewed in [1]).The gene duplication event subsequently results in an increase of both genome size and the entire gene set thereby influencing the architecture and function of many genomes [2, 3]. During the process of adaptation or evolution under reduced selective constraint, duplicated genes acquire novel functions of pre-existing genes [4, 5]. New genes can also arise de novo from intergenic space [6] or new transcriptional regulatory sites on a promoter that alters gene expression [7].The potential *cis*-elements in the promoter regions can also be subject to changes in sequence and specificity in response to developmental stage and environment [8]. Although members of a gene family contain very high sequence identity, their temporal and spatial expression level may differ.

**Fig. 1:**
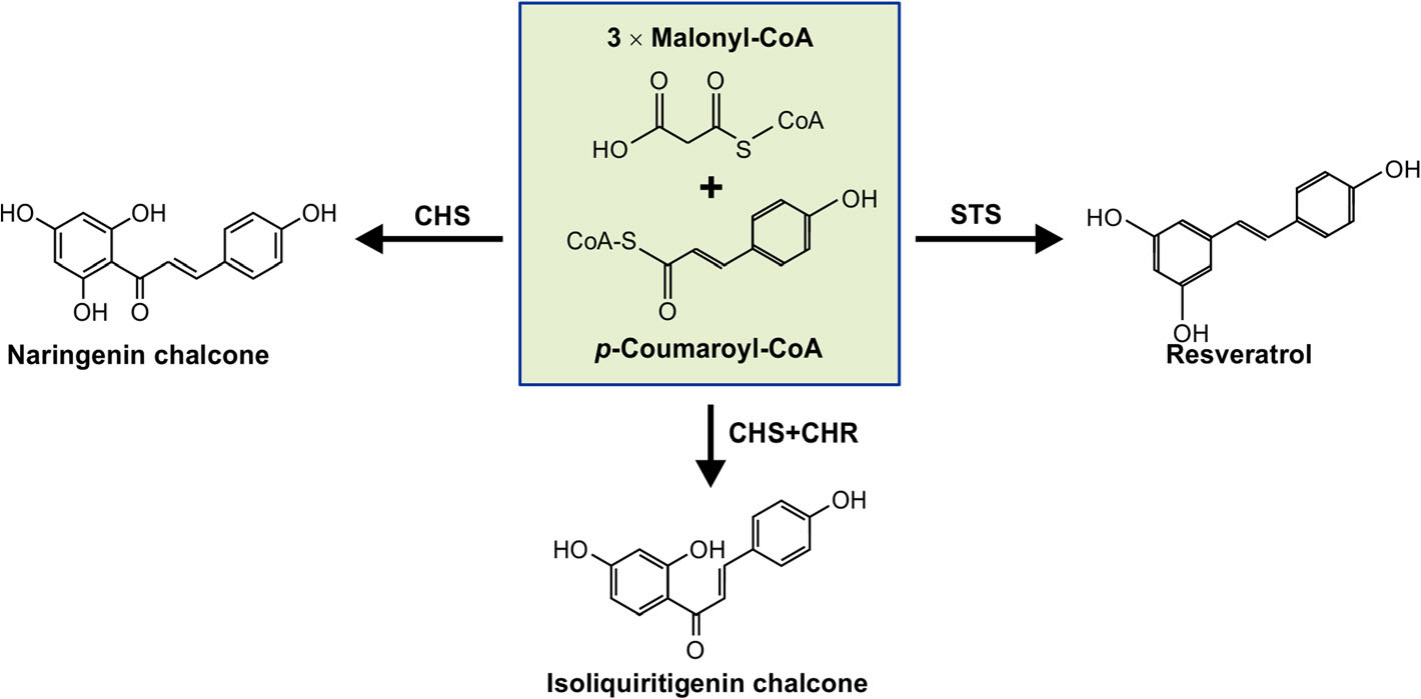
Reactions catalyzed by CHS and STS. Both CHS and STS use the same substrates *p*-coumaroyl-CoA and 3 molecules of malonyl-CoA and convert them to either naringenin chalcone or resveratrol, respectively. In legumes, CHS coacts with a legume-specific enzyme chalcone reductase (CHR) to produce isoliquiritigenin chalcone

Polyketide synthases (PKS) play a critical role in bridging primary and secondary metabolism in plants by catalyzing the sequential condensation of two-carbon acetate units into a growing polyketide chain. PKS enzymes are classified into type I, II, and III based on their catalytic mechanism, domain structure, and subunit organization. While type I and II PKSs are found in bacteria and fungi, type III PKSs are predominantly plant-specific. Type III PKSs act in homodimers, contain a Cys-His-Asn catalytic tetrad in the active site [9–11], and unlike type I and II PKSs, they do not require acyl carrier for their function [12]. These enzymes are known as chalcone synthase (CHS)-like enzymes that include CHS, stilbene synthase (STS), 2-pyronesynthase, acridone synthase, benzophenone synthase, bibenzyle synthase, phlorisovalerophenone synthase, benzalacetone synthase, *C*-methylchalcone synthase, homoeriodictyol/eriodictyol synthase, aloesone synthase, coumaroyltriacetic acid synthase, hexaketide synthase, biphenyl synthase, stilbene carboxylate synthase, octaketide synthase, penta ketide chromone synthase, and anther-specific CHS-like [9]. Among these PKSs, CHS and STS are structurally similar [11, 13], plant-specific and catalyze condensation reactions of *p*-coumaroyl-CoA and 3 acetyl molecules from malonyl-CoA to produce a common tetraketide intermediate which undergoes a claisen condensation reaction catalyzed by CHS [11] or an aldol cyclization catalyzed by STS [14] to give rise to naringenin chalcone and resveratrol, respectively (Fig. 1). In legume plants, CHS co-acts with a legume-specific enzyme, chalcone reductase, to produce isoliquiritigenin chalcone. The production of these chalcones is the first committed step in the biosynthesis of a plethora of (iso)flavonoids, which have been shown to play important roles in protection against various biotic and abiotic stress, flower pigmentation, nitrogen fixation, pollen fertility and seed coat color.

In soybean, seed coat color is one of the important traits for variety development. The *CHS* gene family has been extensively studied as changes in their expression impacts seed coat pigmentation [15]. Soybean is a paleopolyploid that has undergone two whole genome duplication events [16–18] with 75% of genes present in multi-gene families [19]. Earlier, *CHS* superfamily with 9 members was reported in soybean [20]. Here we performed a genome-wide search of *CHS* genes in soybean and identified 21 *GmCHS* loci with 14 unique genes in the genome. In addition to the previously known 9 *GmCHS*, we report 5 new *GmCHS*s in soybean along with their gene architecture, phylogeny, gene expression and protein localization. The results provide evidences towards functional divergence of *GmCHS* that are involved in the production of many important compounds in soybean.

## Results

### The *GmCHS* gene family contains 14 putative members

A first step towards identifying members of *GmCHS* gene family, we used a keyword search ‘chalcone synthase’ within the annotated *G. max* Wm82.a2.v1 genome on Phytozome. This resulted into 1516 genes and 2635 ontologies match. This large number of genes and ontology match was due to the inclusion of all the annotations in the soybean genome database with ‘chalcone’ and/or ‘synthase’. In the list of 2635 ontologies, an ontology with the words ‘chalcone’and ‘synthase’ (PANTHER IDPTHR11877:SF27) was identified which was selected to find other related *GmCHS*s using the ‘shared annotation’ function in Phytozome. This process identified a total of 19 *GmCHS* genes that included previously identified 9 *GmCHSs* [15]. To ensure that all *CHS* genes were identified in soybean, each *GmCHS* was used as a query for a BLAST search which identified two additional *GmCHS*s (Glyma.09G074900 and Glyma.13G034300), making a total of 21 *CHS* loci in soybean genome. Based on the RNAseq data available in the public domain, an expression analysis of *GmCHS* genes was performed. No transcripts for 4 *GmCHS* genes (Glyma.05G153100, Glyma.09G074900, Glyma.11G097900 and Glyma.13G034300) were detected in any tissue suggesting them as pseudogenes. The sequence comparison of the *GmCHS* gene family members revealed that there are three copies of *GmCHS3* (Glyma.08G109300, Glyma.08G110900 and Glyma.08G110300) and two copies of *GmCHS4* (Glyma.08G110700 and Glyma.08G110500). Altogether, we found a total of 14 unique *GmCHS* genes in the soybean genome. These *GmCHS* genes encode proteins with a calculated molecular mass ranging from 37 to 45 kDa. Detailed characteristics of *GmCHS* genes are shown in Table 1.

**Table 1:** List of *GmCHS* genes identified in soybean genome

Gene name	Locus name	Gene location	Coding sequence (nt)	Splice variants	Predicted protein molecular mass (kDa)
Gm*CHS1*	Glyma.08G109400	Chr08: 8391364..8394840	1167	1	45
Gm*CHS2*	Glyma.05G153200	Chr05: 34687009..34693243	1167	1	44
Gm*CHS3a*	Glyma.08G109300	Chr08: 8387509..8391327	1167	1	44
Gm*CHS3b*	Glyma.08G110900	Chr08: 8517799..8519303	1167	2	44
Gm*CHS3c*	Glyma.08G110300	Chr08:8475793..8477410	1167	1	44
Gm*CHS4a*	Glyma.08G110700	Chr08: 8513952..8515719	1167	1	45
Gm*CHS4b*	Glyma.08G110500	Chr08: 8504479..8506020	1167	1	45
Gm*CHS5*	Glyma.08G109200	Chr08: 8384742..8386542	1167	1	45
Gm*CHS6*	Glyma.09G075200	Chr09: 8145494..8147595	1167	1	45
Gm*CHS7*	Glyma.01G228700	Chr01: 55659010..55660950	1170	1	42.8
Gm*CHS8*	Glyma.11G011500	Chr11: 802453..804663	1170	2	42.8
Gm*CHS9*	Glyma.08G109500	Chr08: 8397944..8399751	1167	1	44
Gm*CHS10*	Glyma.02G130400	Chr02: 13399253..13401493	1167	1	45
Gm*CHS11*	Glyma.01G091400	Chr01: 27621455..27623628	1167	1	44
Gm*CHS12*	Glyma.08G110400	Chr08: 8478834..8480215	1023	1	37
Gm*CHS13*	Glyma.19G105100	Chr19: 35466392..35469297	1176	1	43
Gm*CHS14*	Glyma.06G118500	Chr06: 9644661..9650144	1170	1	43

An alignment of deduced protein sequences of GmCHSs revealed very high sequence identity in the entire region. Among the GmCHSs, GmCHS14 was most diverse and showed only 43 to 52.9% sequence identity at amino acid level with other GmCHS isoforms. Pairwise percentage identity of other GmCHSs at amino acid and nucleotide levels varied from 73.4 to 100% and 67.7 to 100%, respectively (Additional file 1: Table S1). Since there are 3 copies of *GmCHS3* and 2 copies of *GmCHS4*, we analyzed the promoter regions (1000 bp upstream of translational start site) of all *GmCHS*s. A pairwise sequence comparison between all candidate gene promoters showed sequence identity ranging from 0.4 to 100% (Additional file 2: Table S2). Even though coding region DNA sequence identities between the 3 copies of *GmCHS3* range from 99.9 to 100%, their promoter sequence differ significantly (2.6 to 48.9% identity). Therefore, we named the 3 copies of *GmCHS3* as *GmCHS3a* (Glyma.08G109300)*, GmCHS3b* (Glyma.08G110900), and *GmCHS3c* (Glyma.08G110300). Similarly, the promoter sequences of two copies of *GmCHS4* are 81.6% identical, were named as *GmCHS4a* (Glyma.08G110700) and *GmCHS4b* (Glyma.08G110500). Despite that *GmCHS5* and *GmCHS12* coding region sequences only share 87.4% identity, their promoter regions (upto 1000 bp upstream of translational start site) contain 100% identical sequence.

### Sequence comparison and phylogenetic analysis of GmCHS

**Fig. 2:**
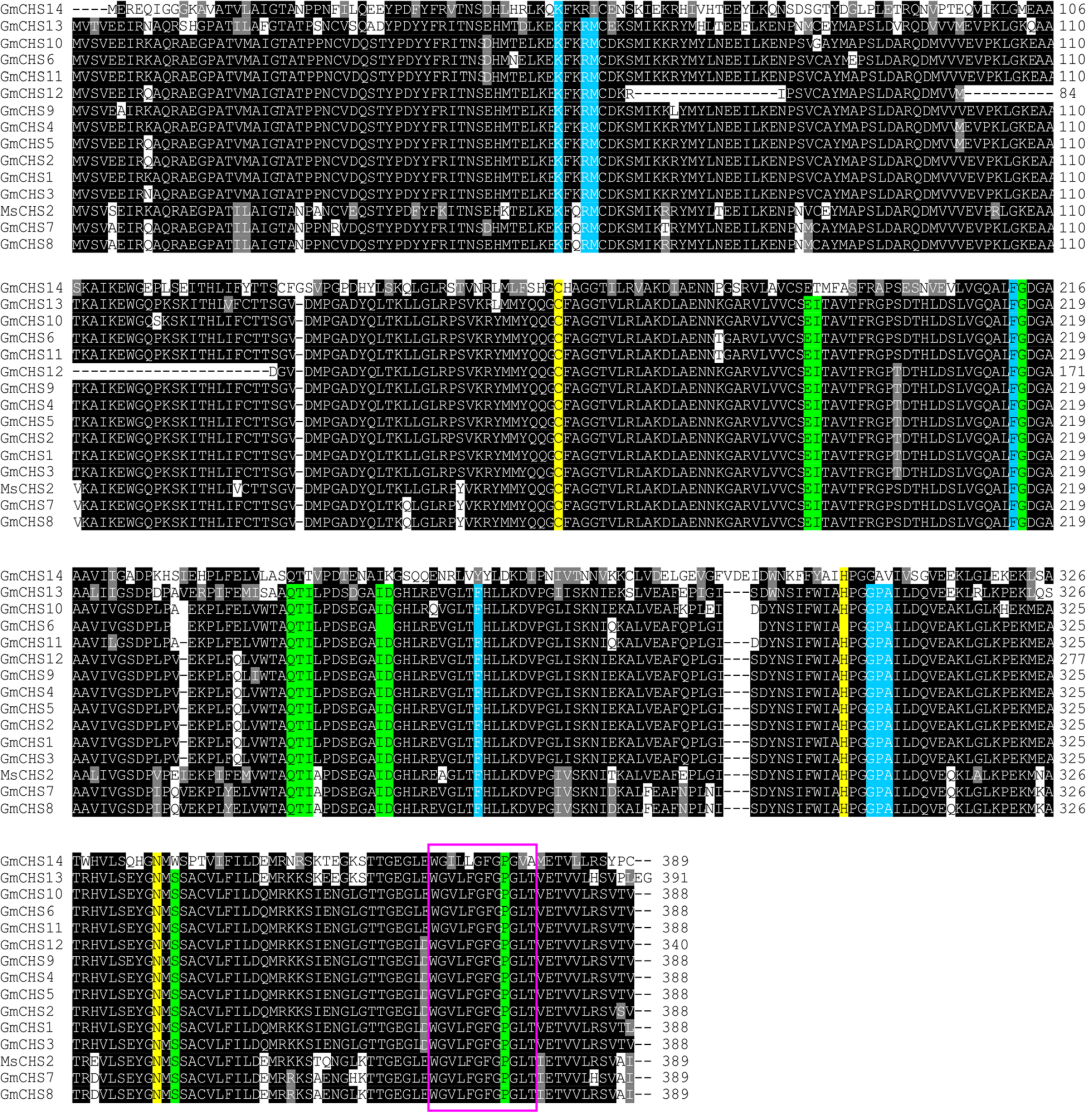
Analysis of deduced aminoacid sequences of GmCHSs. Multiple sequence alignment of amino acid sequences of GmCHSs and CHS2 from alfalfa (MsCHS2) were performed using ClustalΟ. Identical residues are shown in black and similar residues are in grey. A hyphen indicates a gap. Active site residues are highlighted in yellow, malony-CoA binding sites are highlighted in blue and product binding residues are shown in green. The characteristic CHS signature (WGVLFGFGPGLT) is indicated by a red box

The crystal structure of CHS from *Medicago sativa* (MsCHS2) has elucidated the importance of four active site residues (Cys 164, Phe 215, His 303 and Asn 336) where the Cys-His-Asn triad is critical for substrate binding [11]. To evaluate if GmCHSs contain the active site residues and CHS/STS signature motif (WGVLFGFGPGLT), we performed a sequence alignment of all putative GmCHSs using their deduced amino acid sequence with MsCHS2. The result revealed that the PKS type III active sites of the enzymes are conserved among all 14 GmCHS (Fig. 2). The CHS/STS signature motif was conserved in all GmCHSs except for GmCHS14 where four amino acid substitutions (V369I, F371 L, L377 V and T278A) were found. The product and malonyl-CoA binding sites are also present in all GmCHS proteins except GmCHS14. These findings suggest that GmCHS14 may have a different function than its isoforms. Furthermore, GmCHS12 contains all the critical residues necessary for CHS, but it has 3 large deletions within its sequence.

**Fig. 3:**
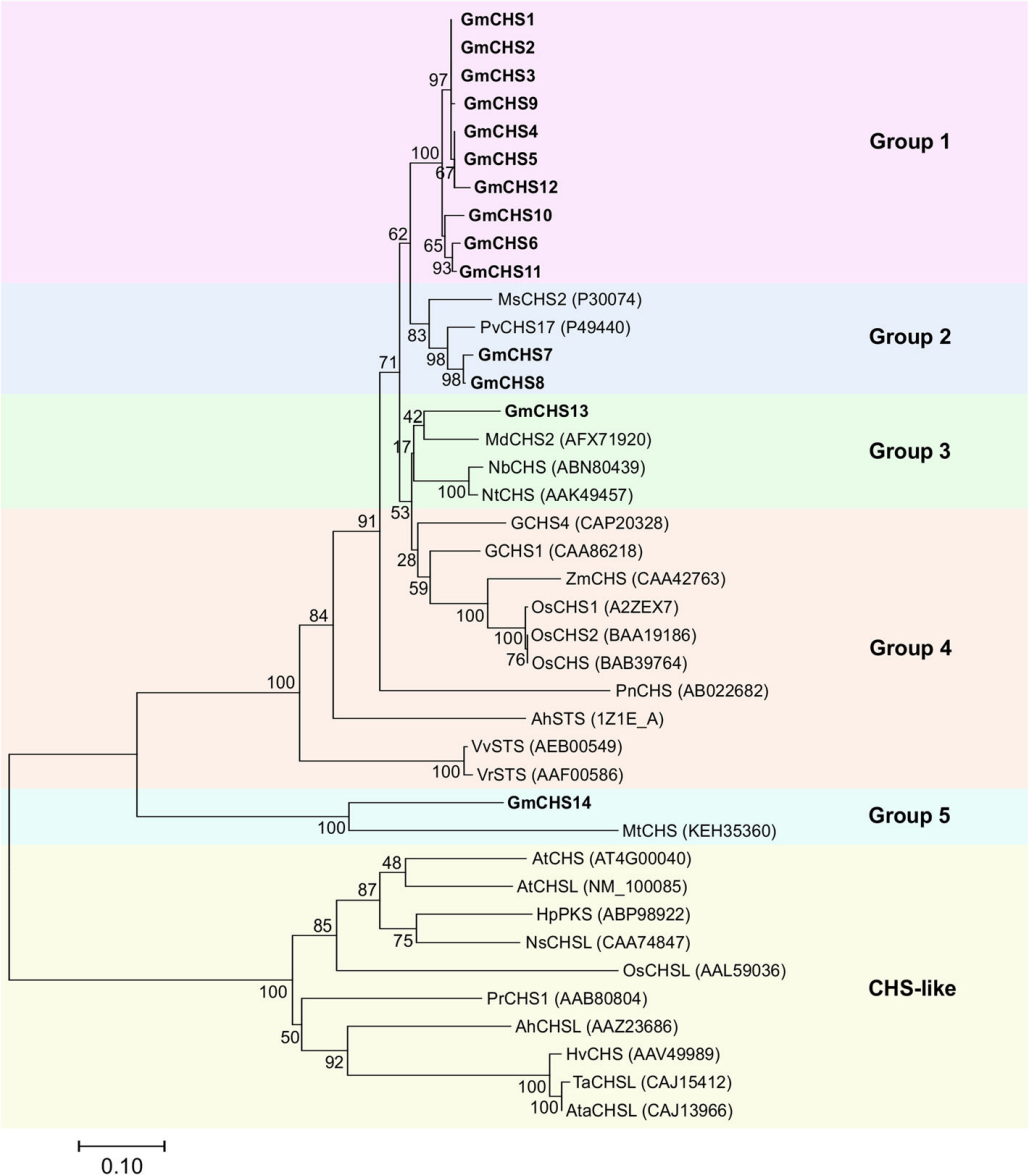
Molecular phylogenetic analysis of the deduced amino acid of GmCHS. The deduced amino acid sequences of the GmCHSs from soybean were aligned with characterized CHS and CHS-like proteins from other plant species and the evolutionary tree was generated using the Neighbor-Joining method in MEGA7 [45]. The percentage of replicate trees in which the associated taxa clustered together in the bootstrap test is shown next to the branches. Scale bar indicates branch length representing residue substitution per site. GmCHS are indicated in bold. At, *Arabidopsis thaliana*; Os, *Oryza sativa*; Ms., *Medicago sativa*; Mt., *Medicago truncatula*; Md, *Malus domestica*; Pv, *Phaseolus vulgaris*; Vr, *Vitis riparia*; Vv, *Vitis vinifera*; Ah, *Arachis hypogaea*; Pr*,Pinu sradiata*; Hp, *Hypericum perforatum*; Ns, *Nicotiana sylvestris*; Hv, *Hordeum vulgare*; Ta,*Triticum aestivum*; Ata, *Aegilops tauschii*; AhCHL, *Arabidopsis halleri;* Pn, *Psilotum nudum*; Nb, *Nicotiana benthamiana*; Nt, *Nicotiana tobaccum*; Zm, *Zea mays*

To elucidate the evolutionary relationship within GmCHS isoforms and with CHS from other plant species, we performed a phylogenetic analysis by comparing the amino acid sequences of 14 putative GmCHSs along with previously characterized CHS, CHS-like and STS proteins from other plant species. As shown in Fig. 3, GmCHSs clustered into 4 distinct groups. Group 1 consisted of 10 GmCHSs where 6 of them (GmCHS1, GmCHS2, GmCHS3, GmCHS9, GmCHS4 and GmCHS5) are tightly clustered, and except for GmCHS2, other 5 GmCHSs reside on chromosome 8. Group 2 contained GmCHS7 and GmCHS8 which formed a close clade with previously characterized legume-specific CHSs, PvCHS17 and MsCHS2. Group 3 and group 5 contained GmCHS13 and GmCHS14, respectively. GmCHS14 was much closer to STS from *Vitis riparia*, *V. vinifera*, and *Arachis hypogaea* in the evolutionary tree. CHSs from monocots such as rice (OsCHS1, OsCHS2, and OsCHS3) and maize (ZmCHS) formed a separate clade (group 4)*.* CHS-like proteins from different species including Arabidopsis CHS formed a distinct clade from most of the known CHS in the phylogenetic tree demonstrating the divergent of CHS super family in plants.

To determine the selective evolutionary pressure on the divergence of *GmCHS* genes, we obtained 40,972 duplicated genomic regions with non-synonymous (Ka) and synonymous (Ks) values for each duplicated gene pairs in soybean genome from Plant genome duplication database (Additional file 3: Table S3). Extraction of duplicated *GmCHS* genes from the list of 40,972 genes led to 3 duplicated *GmCHS* gene pairs: i) *GmCHS5* and *Glyma.05G153100* (pseudogene), ii) *GmCHS7* and *GmCHS8* and iii) *GmCHS10* and *GmCHS11* (Table 2). Genes with purifying selection during evolution have Ka/Ks value less than 1. The Ka/Ks values for the duplicated *GmCHS* gene pairs ranged from 0.065 to 0.549 (Table 2) indicating that they may have acquired limited functional divergence following the duplication events.

**Table 2:** Estimated Ka/Ks values of duplicated *GmCHS* genes in soybean

E_Value	Locus_1	Locus_2	Ka	Ks	Ka/Ks	% Identity
3.00E-64	Glyma.05G153100 (Pseudogene)	Glyma.08G109200 (*GmCHS5*)	0.196	0.357	0.549	32.1
0	Glyma.01G228700 (*GmCHS7*)	Glyma.11G011500 (*GmCHS8*)	0.008	0.083	0.094	88.5
0	Glyma.01G091400 (*GmCHS11*)	Glyma.02G130400 (*GmCHS10*)	0.011	0.176	0.064	83.5

### Chromosomal arrangement and gene structure of *GmCHSs*

**Fig. 4:**
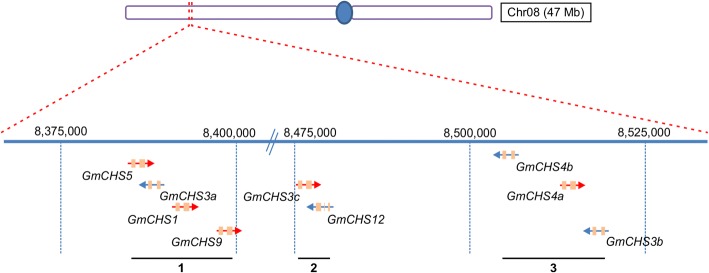
Schematic diagram showing *GmCHS* gene clusters on chromosome 8. A 135 kb gene rich region of chromosome 8 showing *GmCHS* gene clusters (cluster 1–3) is shown. Arrows represent each *GmCHS* locus. Red and blue arrows indicate the *GmCHS* genes in ‘+’ and ‘-’ strand, respectively drawn to scale. Numbers on the chromosome are in bp units

The 21 *GmCHS*s including 14 unique genes and 3 duplicate copies are distributed on 8 different chromosomes in soybean. Gene density in these 8 chromosomes is even (one gene per chromosome) except for chromosome 1 and 8 which contain 2 and 9 *GmCHS* genes, respectively (Table 1). The 9 *GmCHS* genes on chromosome 8 are located within a 135 kb gene rich region that contained a total of 18 genes. As shown in Fig. 4, the 9 *GmCHSs* on chromosome 8 form 3 distinct clusters with each cluster containing a copy of *GmCHS3.* Cluster 1 contains *GmCHS5, GmCHS3a, GmCHS1* and *GmCHS9* within a 15 kb region where they are arranged in tail to tail, head to head or head to tail orientations. Cluster 2 contains *GmCHS3c* and *GmCHS12* arranged tail to tail. Lastly, a 14.8 kb region at the location 8,504,479..8519303 on chromosome 8 forms cluster 3 that contains 2 copies of *GmCHS4* (*GmCHS4b* and *GmCHS4a*) arranged in the head to head orientation and *GmCHS3b.* Detailed information on all 18 genes within the 135 kb region on chromosome 8 is shown in Additional file 4: Table S4.

**Fig. 5:**
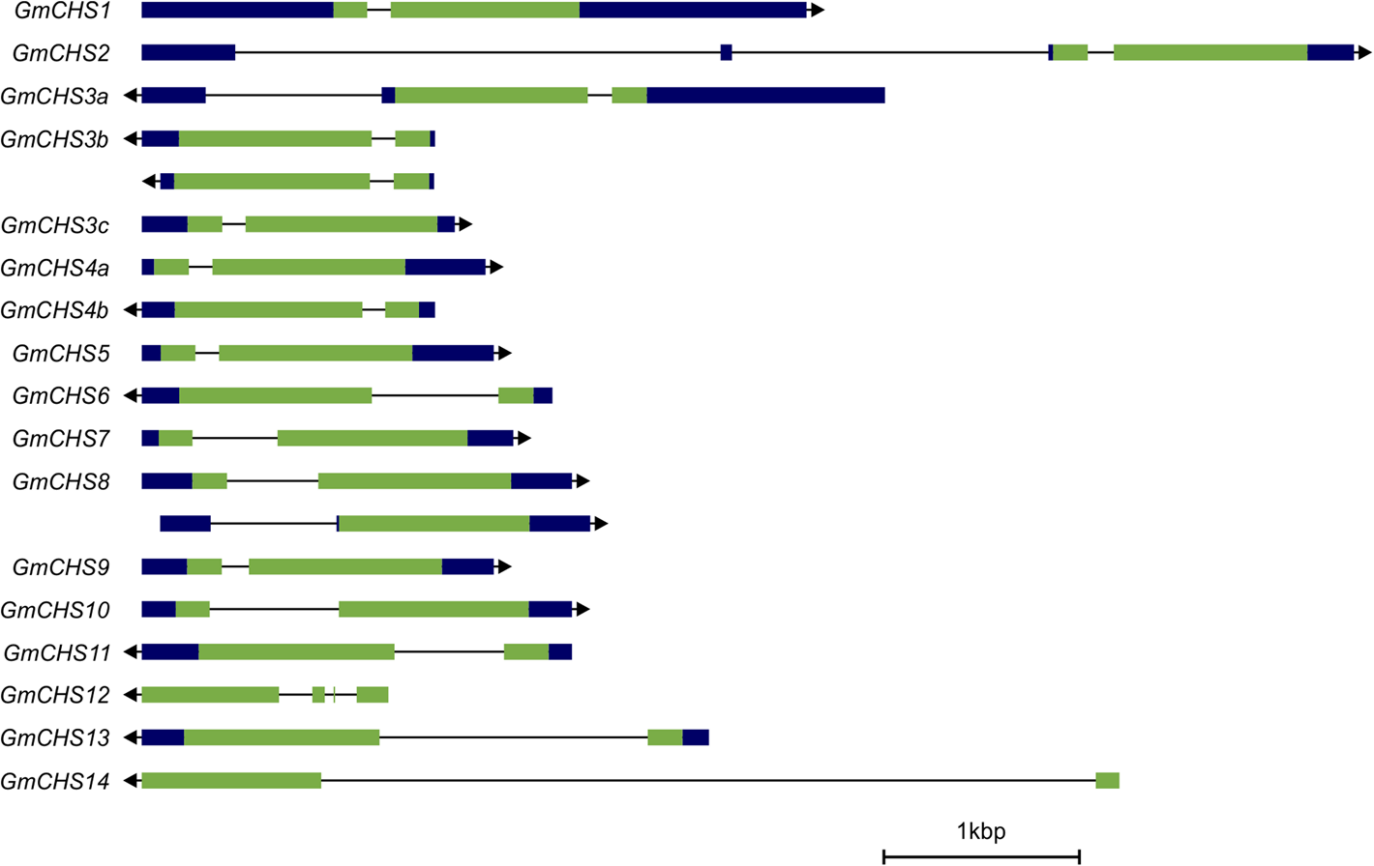
Schematic diagrams of *GmCHS* gene structures. *GmCHS* gene structures with predicted alternate transcripts were compiled from Phytozome database (https://phytozome.jgi.doe.gov/pz/portal.html#!info?alias=Org_Gmax). The black and green boxes represent UTRs and exons, respectively, while lines indicate introns. Right pointing arrows indicate ‘+’ strand while left pointing arrows indicate ‘-’ strand, relative to the genome sequence. Gene structure images are drawn to scale as indicated

Analysis of gene structure of *GmCHS* genes revealed 2 exons and 1 intron except for *GmCHS12* that contained 4 exons and 3 introns (Fig. 5). Even though the majority of *GmCHS*s contained a single intron, their intron size varied within the family members ranging from 121 to 4347 nucleotides. Additionally, the presence of a single intron in *GmCHS3a* 3’UTR and 2 introns in *GmCHS2* 5’UTR was found.

### Expression analysis of *GmCHS* gene family

**Fig. 6:**
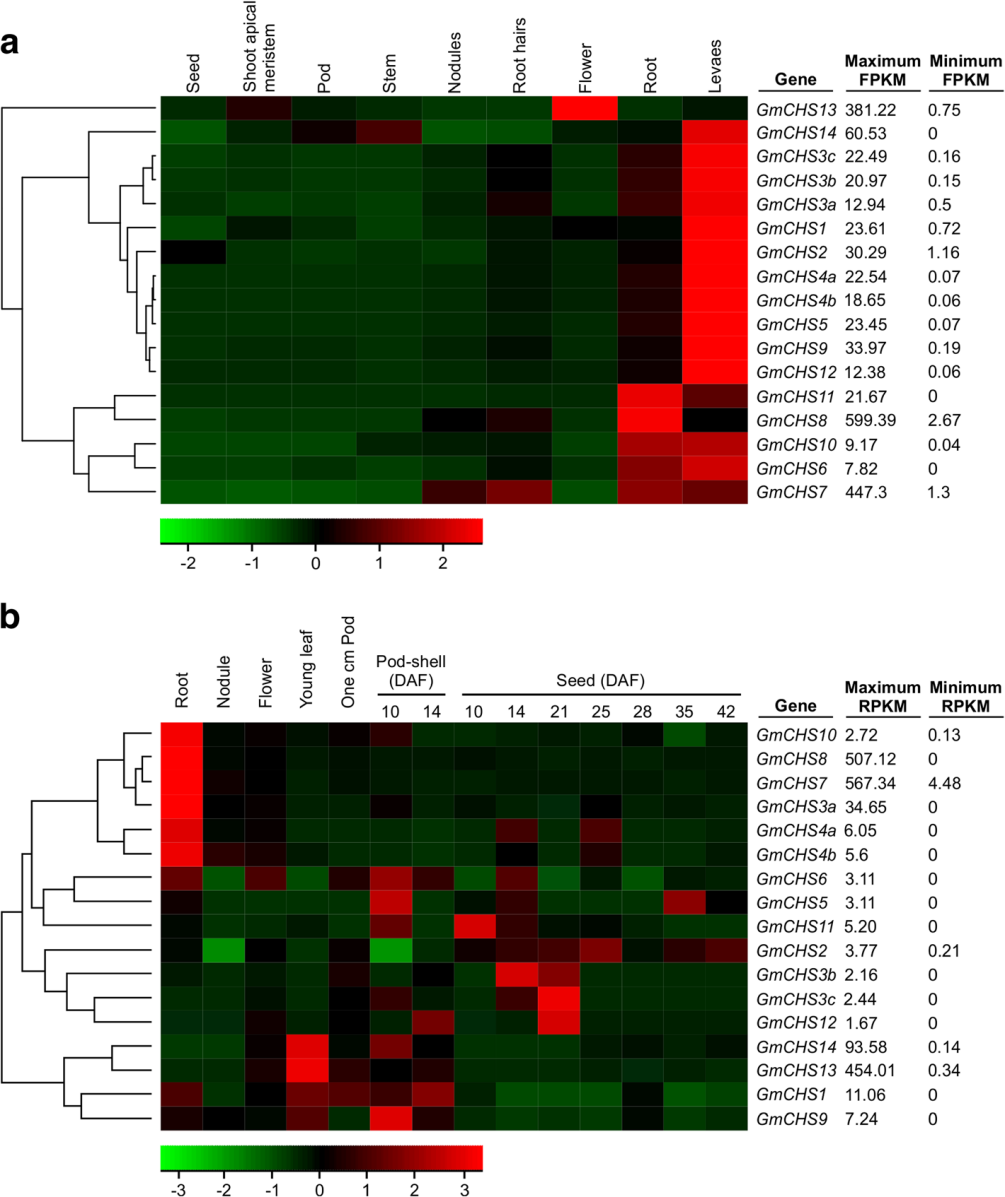
Tissue-specific expression profile of the *GmCHS* gene family. The transcriptome data of *GmCHS* genes in soybean across different tissues were retrieved from **a** Phytozome database [21], and **b** Soybase database [23] for heatmap generation. The color scale indicates expression values, green indicating low transcript abundance and red indicating high levels of transcript abundance. Maximum and minimum FPKM or RPKM value for each gene is shown

To determine the tissue-specific gene expression patterns of the *GmCHS* gene family, we used two sets of the publicly available genome-wide transcript profiling data of soybean tissue as a resource [21–23]. The Libault et al. [21] dataset consisted of the transcript abundance in soybean tissues such as flower, shoot apical meristem, seed, pod, stem, root, nodule, leaf, and root hair. Fragments perkilobase of transcript per million mapped reads (FPKM) values of *GmCHS*s in their highly expressed tissues varied from 7.82 to 599.39 (Additional file 5: Table S5). As shown in Fig. 6a, the majority of the *GmCHS*s were highly expressed in leaves. Transcripts of *GmCHS6, GmCHS7, GmCHS8, GmCHS10* and *GmCHS11* were abundant in roots compared to other tissues. Accumulation of *GmCHS13* transcript was higher in flowers compared to other tissues. The expression patterns of three copies of *GmCHS3* displayed differential expression patterns in soybean tissues while the two copies of *GmCHS4* showed almost similar expression patterns. Based on the transcriptome data, no expression of *GmCHS14* was observed in nodules, while no expression of *GmCHS6*, *GmCHS11* and *GmCHS14* was observed in seed tissue. The second dataset by Severin et al. [23] consisted of the transcript abundance in soybean tissues such as root, flower, young leaf, nodule, and pods and seeds at several different developmental stages. Reads per kilobase of transcript per million mapped reads (RPKM) values of *GmCHS*s in their highly expressed tissues varied from 1.673 (*GmCHS12* in seed 21-DAF) to 567.342 (*GmCHS7* in roots) (Additional file 5: Table S5). As this study included pod and seed tissue samples at multiple stages of development, it provided a better assessment of expression levels of *GmCHS* genes in seed tissue compared to the earlier study (compare Fig. 6a and b). In both the datasets, transcripts of *GmCHS7, GmCHS8,* and *GmCHS10* were abundant in roots compared to other tissues. Similarly, *GmCHS1, GmCHS9* and *GmCHS14* transcripts accumulated at higher levels in leaf tissue. However, some differences in expression patterns of *GmCHS*s were observed in these two sets of studies. For example, relative transcripts abundance for *GmCHS3a, GmCHS4a* and *GmCHS4b* in root tissue did not match in these two studies (Fig. 6a and b).

**Fig. 7:**
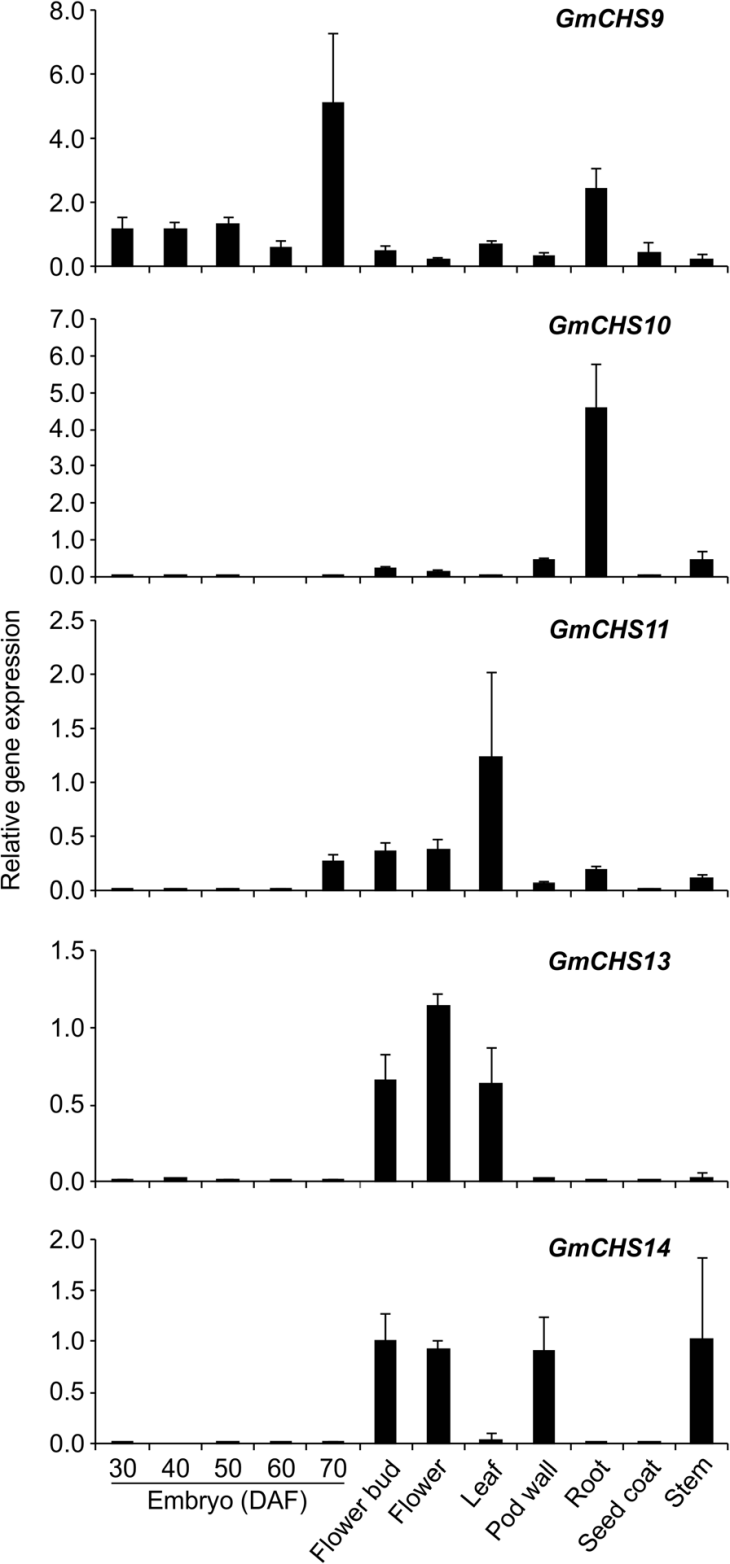
Expression analysis of five *GmCHS* genes in soybean tissues. Total RNA (1 μg) from soybean root, stem, leaf, flower bud, flower, pod wall, seed coat and embryos (30, 40, 50, 60 and 70 DAF) was used for cDNA synthesis and qPCR using gene-specific primers. Error bars indicate SEM of two biological replicates, with three technical triplicates. Values were normalized against the reference gene *CONS4*

To validate the RNAseq expression data, we studied the tissue-specific expression of newly discovered *GmCHS*s along with *GmCHS9* by qRT-PCR. RNA isolated from vegetative and reproductive tissues of soybean during the development was subjected to qRT-PCR analysis. Our results correlate with the two previously reported RNAseq studies. As shown in Fig. 7, expression of *GmCHS10* was abundant in roots and *GmCHS13* in flowers which correlate with the RNAseq data (Fig. 6). A low expression of *GmCHS10, GmCHS11, GmCHS13,* and *GmCHS14* was observed in embryo tissues (30 to 70 DAF) (Fig. 7) and results are consistent with the RNAseq study. Despite that Fig. 6b contained expression of *GmCHS* genes in seed tissues, only two developmental stages of seeds (28 and 42 DAF) were closer to embryo (30 and 40 DAF) used in our study. To determine the expression divergence of duplicated genes, the gene expression values of the samples (root, nodule, and flower) common in the two publically available RNAseq datasets [21, 23] were analysed by type II one-way ANOVA followed by multiple comparison post hoc Tukey’s test. The results revealed that the expression pattern of *GmCHS7* and *GmCHS8* duplicated pairs were significantly different than the other *GmCHS* genes in root, nodule, and flower tissues. However, no such difference was identified for other two duplicated *GmCHS *gene pairs.

### Subcellular localization of GmCHS isoforms

**Fig. 8:**
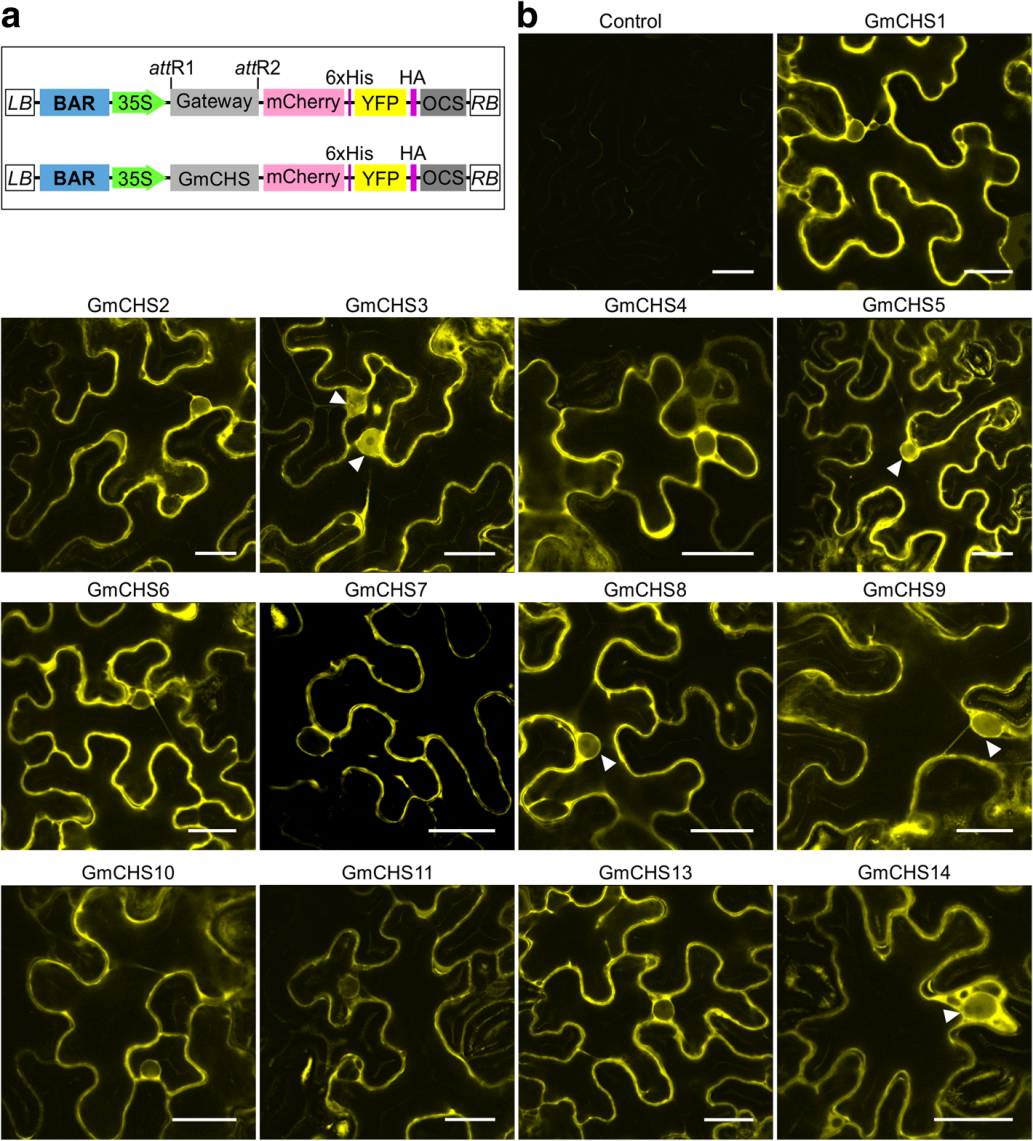
Subcellular localization of GmCHS family *in planta*. **a** A schematic diagram showing double reporter expression vector. **b** The *GmCHS* genes were translationally fused upstream of the dual reporter genes, mcherry and YFP, transformed into *N. benthamiana* by *Agrobacterium* mediated transformation and visualized by confocal microscopy. Nuclear localization of GmCHSs are shown by white arrow heads. An empty vector control is also included. Scaler bar indicates 25 μm

Previously we reported the dual subcellular localization (cytoplasm and nucleus) of GmCHS8 [24]. Even though all GmCHS isoforms were predicted to be cytosolic, we determined their localization *in planta*. A translational fusion of full-length *GmCHS* was created upstream of the dual reporter genes mCherry and YFP (Fig. 8a), and transiently expressed in leaf epidermal cells of *N. benthamiana*. Attempts to clone *GmCHS12* were not successful due to its low level of expression. Therefore, GmCHS12 was not included in the subcellular localization study. To avoid the passive diffusion of GmCHS proteins to the nucleus, a dual reporter vector was created by adding mCherry in the vector pEarlygate101 which increased the size of the fusion protein. As shown in Fig. 8b, all 13 GmCHS proteins were observed in the cytoplasm. Additionally, 5 GmCHSs (GmCHS3, GmCHS5, GmCHS8, GmCHS9 and GmCHS14) were also found in the nucleus.

## Discussion

Plant genomes tend to evolve faster than mammals resulting into more dynamics and higher genome diversity [25]. Large plant genome with multi-gene families results from multiple factors such as gene duplication, whole genome duplication and domestication. Most plant species contain small *CHS* gene families. For example, Arabidopsis genome contains a single *CHS* gene [26] while *Petunia hybrida* [27], *Ipomea purpurea* [28], *Gerbera hybrida* [29] and *Pisum sativum* [30] contain 8, 6, 3 and 8 *CHS* members, respectively. Recently, a *CHS* gene family containing 14 members were identified in maize [31]. Here we have identified a total of 14 unique *CHS* genes (*GmCHS1-GmCHS14*) in the soybean genome. Our genome-wide search in soybean revealed 21 CHS loci that included 3 copies of *GmCHS3*, 2 copies of *GmCHS4* and 4 pseudogenes. The *I* locus that controls the seed coat color in soybean was previously described to contain two identical clusters (tandem inverted repeats) of *CHS1*, *CHS3* and *CHS4* [20]. Such tandem repeats were not found in our analysis of *Glycine max Wm82.a2.v1*. However, the *CHS* gene rich region on chromosome 8 contained 9 *CHS* loci, 5 on the sense strand and 4 on the antisense strand (Fig. 4). Many of the *GmCHS* gene family members contain very high sequence identity. For example *GmCHS4* and *GmCHS5* share 99.7% sequence identity at the nucleotide level (Additional file 2: Table S2). It is possible that with such a high sequence identity, together with *GmCHS* gene organization in the chromosome, this may lead to the inverted repeats and give rise to mutations in the *I* locus [32].

Members of *CHS* gene family in other species showed functional variations and tissue-specific expression patterns, for example, among three *CHS* genes that showed different spatial and temporal regulation in *Gerbera hybrida*, only *GCHS1* contributing to flavonoid biosynthesis [29]. Since CHS and STS use the same substrate and the catalytic active sites area consensus among these proteins, the involvement of these enzymes in either flavonoid or stilbene biosynthesis will not be known until enzyme activity assays are conducted.

CHS forms a homodimer for its enzymatic activity. The CHS homodimer contains two functionally independent active sites. CoA-thioesters and product analogs occupy both active sites of the homodimer in the CHS complex structures. These structures identify the location of the active site at the cleft between the lower and upper domains of each monomer, where few chemically reactive residues are present in the active site [11, 33]. The four conserved amino acid residues, specifically Cys 164, Phe 215, His 303 and Asn 336 (numbering based on MsCHS2), which form active sites in all CHS-related enzymes [11] are conserved among the GmCHS isoforms (Fig. 2). Cys 164 serves as the nucleophile and as the attachment site for polyketide intermediates in both CHS and STS. The nitrogen electron of His 303 is within hydrogen-bonding distance of the sulfur atom of Cys 164 and His 303 most likely acts as a general base during the generation of a nucleophilic thiolate anion from Cys 164. The active site architecture of CHS consists of three interconnected cavities that intersect with these four residues and these cavities include a coumaroyl-binding pocket, CoA-binding tunnel, and a cyclization pocket [11]. Since GmCHS14 sequence differs mostly from other GmCHSs, and it lacks important residues that affect binding and cyclization pockets, it may function differently and may catalyze a different reaction.GmCHS7 and GmCHS8 are possibly the active CHSs in soybean as they share the same clade with PvCHS17 and MsCHS2 in the phylogenetic tree (Fig. 3). *GmCHS12* contains several deletions within the sequence and produces a protein with lower molecular mass compared to its other isoforms (Table 1). However, the critical residues necessary for its activity are conserved in GmCHS12 suggesting it may be functionally active.

Gene family members with variation in the *cis*-architecture of a promoter DNA region result in differential expression patterns within a species. Members of *CHS* gene family in *Gerbera hybrida* showed functional variations and tissue- and development-specific expression patterns [29]. Despite that both *GCHS1* and *GCHS4* are expressed in gerbera petals, only GCHS1 is responsible for flavonoid biosynthesis in gerbera petals while GCHS4 has a role in pigment production in vegetative tissues. Most of the *GmCHS* transcripts accumulate abundantly in soybean leaves and roots suggesting their importance in these tissues. The expression of these genes in soybean roots is highly important since downstream of the CHS-catalyzed step is the production of isoflavonoids that participate in plant defense mechanisms, and also in the symbiotic relationship between soybean and bacteria for nitrogen fixation. The high expression of *GmCHS7* and *GmCHS8* in soybean tissues have already been studied [34] which is consistent with the expression analysis reported here (Fig. 6). Most *GmCHS*s were expressed in soybean leaves and roots which could explain the requirement of these genes in the respective tissues for (iso)flavonoid biosynthesis. Diverse expression of *GmCHS* genes in soybean tissues may be due to their diverse promoter regions except for *GmCHS5* and *GmCHS12* as their promoters are 100% identical (Additional file 3: Table S3). Identical promoter regions with conserved *cis*-regulatory elements could be a result of segmental duplication and it has been observed previously among certain duplicated genes [34]. Gene family members showing diverse gene expression in soybean have been documented. For example, soybean 14–3-3 protein (*SGF14*s) [35], *GmCHR* [36] and *chalcone isomerase* (*GmCHIs*) [37] family members also display differential expression patterns in soybean tissues.

Our findings that GmCHS isoforms localize to the cytoplasm and nucleus adheres to the co-localization of other (iso)flavonoid enzymes [36–38] and isoflavonoid metabolon [24]. Since (iso)flavonoid biosynthesis involves multiple cytochrome P450s that are ER localized and are not in the nucleus, the presence of some GmCHS family members raises the possibility of additional role of these enzymes in the nucleus.

## Conclusion

Overall, we have performed a comprehensive analysis of *CHS* genes present in soybean genome and identified 14 unique *GmCHS*s where 6 of them along with copies of *GmCHS3* and *GmCHS4* reside on chromosome 8 in 3 separate clusters. Based on the phylogenetic analysis, GmCHS13 and GmCHS14 are distantly related to other GmCHSs suggesting their diverse roles. Furthermore, temporal and spatial expression of *GmCHS* members and GmCHS isoform specificity at a sub-cellular level shed light on alternative function of some isoforms.

## Methods

### Plant material

*Nicotiana benthamiana* seeds were obtained from Dr. Rima Menassa (London Research and Development Centre, Agriculture and Agri-Food Canada). Seeds were grown in a growth room under a 16 h light/8 h dark cycle at 25 °C/20 °C with relative humidity of 60–70%. For transient expression, the intact leaves of 6 to 8-week old *N. benthamiana* plants were used.

### In silico and phylogenetic analysis

To identify putative *GmCHS* genes in soybean, the Phytozome database (https://phytozome.jgi.doe.gov/pz/portal.html) [22] was used for a keyword search using ‘chalcone synthase’ in the annotated *G. max* Wm82.a2.v1 genome. Each *CHS* identified in the soybean genome was used as a query for a nucleotide BLAST (BLASTn) search. Protein sequences were retrieved for all GmCHSs and their calculated molecular mass was determined using the web-based tool ExPASy (https://web.expasy.org/translate/). Prediction of subcellular localizations was performed using TargetP (http://www.cbs.dtu.dk/services/TargetP/) with default parameters. Duplicated genomic regions and Ka/Ks values for each duplicated genes in soybean genome was obtained from Plant Genome Duplication Database (http://chibba.agtec.uga.edu/duplication/).The duplicated *GmCHS* gene pairs were extracted manually from the list of duplicated genes in soybean genome.

For phylogenetic analysis, the amino acid sequences were aligned in ClustalΟ and a Neighbour-joining tree was constructed with 1000 bootstrap replications by using MEGA7 [39]. Pairwise nucleotide and amino acid comparison were performed using the sequence identity matrix function in BioEdit Sequence Alignment Editor Version 7.5. Active sites, malonyl-CoA binding sites and product binding sites on sequences of GmCHSs were identified using NCBI conserved domain search (https://www.ncbi.nlm.nih.gov/Structure/cdd/wrpsb.cgi).

### Generation of a heat map

Two sets of RNAseq data from different soybean tissues are publically available and the expression values are presented in fragments per kilobase of transcript per million mapped reads (FPKM) or reads per kilobase of transcript per million mapped reads (RPKM). FPKM values of all *GmCHS*s in soybean tissues were retrieved from Phytozome (https://phytozome.jgi.doe.gov/pz/portal.html) [22]. Raw data for the second set of RNAseq experiment was downloaded from https://www.soybase.org/ [23]. Reads were trimmed, mapped to the soybean reference genome and RPKM values were calculated in CLC genomic workbench (Qiagen, USA).Heatmaps for expression levels of *GmCHS*s in soybean tissues were generated in R using the heatmap.2 function from the gplots library. The gene expression values for root, pod and flower tissues from two sets of data were used for expression divergence analysis by type II one-way ANOVA followed by multiple comparison post hoc Tukey’s test.

### Quantitative RT-PCR analysis

For qRT-PCR studies, RNA was isolated from 12 different soybean tissues according to Wang and Vodkin [40]. Total RNA (1 μg) was reverse transcribed using the ThermoScript™RT-PCR System (Invitrogen, USA). Gene-specific primers sequences for qPCR are in listed in Additional file 6: Table S6. All reactions were performed in three technical replicates, and the expression was normalized to the reference gene *CON4* [41]. The experiment included two biological replicates. The data were analyzed using CFX manager (BioRad, USA).

### Plasmid construction and subcellular localization

For subcellular localization study, *GmCHSs* were amplified from soybean cDNA by PCR using gene-specific primers. Primers used for *GmCHS*s amplification are listed in Additional file 6: Table S6. The PCR products were cloned into the gateway entry vector pDONR-Zeo (Invitrogen) using BP clonase (Invitrogen), followed by transformation into *Escherichia coli* DH5α. The recombinant plasmid pDONZ-GmCHS was sequence confirmed and recombined with the destination vector pEGmCherry101using LR clonase reaction mix (Invitrogen). The recombinant plasmids were transformed into *Agrobacterium tumefaciens* GV3101 via electroporation. To create pEGmCherry101, mCherry fragment was amplified by PCR using primers AvrII-mCherry-F and XbaI-6His-mCherry-R (Additional file 6: Table S6). The resulting PCR products were digested with AvrII and XbaI, and inserted into the AvrII site at the N-terminus of the YFP in pEarleyGate101 [42].

The pEGmCherry-GmCHS constructs in *A. tumefaciens* GV3101 were transformed into *Nicotiana benthamiana* leaf by infiltration [43] and transient expression was visualized through a Leica TCS SP2 inverted confocal microscope. For confocal microscopy, a 63X water-immersion objective was used at excitation wavelengths at 514 nm and emission spectra of 530-560 nm for YFP.

## Additional files

Additional file 1: Protein and coding DNA sequence identity matrix of GmCHSs. (DOCX 24 kb)

Additional file 2: Promoter sequence identity matrix of *GmCHS* genes. (DOCX 20 kb)

Additional file 3: List of 40,972 duplicated gene pairs in *Glycine max* genome (XLSX 2404 kb)

Additional file 4: List of genes within 134.56 kb region containing *GmCHS* on chromosome 8 in soybean. (DOCX 19 kb)

Additional file 5: *GmCHS* transcript abundance in soybean tissues. (XLSX 17 kb)

Additional file 6: Sequences of oligonucleotides used in the study. (DOCX 24 kb)

## Abbreviations

CHS: Chalcone synthase
FPKM: Fragments perkilobase of transcript per million mapped reads
PKS: Polyketide synthase
RPKM: Reads per kilobase of transcript per million mapped reads
