# Supplementary material for: Genome-wide identification and localization of chalcone synthase family in soybean (Glycine max [L]Merr)
Source: BMC Plant Biol. 2018 Dec 4;18:325. doi: 10.1186/s12870-018-1569-x (PMC6278125; doi:10.1186/s12870-018-1569-x)
Supplement: Supplementary file 1 — Protein and coding DNA sequence identity matrix of GmCHSs. (DOCX 24 kb) [file 12870_2018_1569_MOESM1_ESM.docx]

Table S1. Protein and coding DNA sequence identity matrix of *GmCHS*s.

|  | | **Amino acid (% identity)** | | | | | | | | | | | | | | | | |
| --- | --- | --- | --- | --- | --- | --- | --- | --- | --- | --- | --- | --- | --- | --- | --- | --- | --- | --- |
|  |  | CHS1 | CHS2 | CHS3a | CHS3b | CHS3c | CHS4a | CHS4b | CHS5 | CHS6 | CHS7 | CHS8 | CHS9 | CHS10 | CHS11 | CHS12 | CHS13 | CHS14 |
| **Nucleotide (% identity)** | *CHS1* |  | 99.2 | 99.4 | 99.4 | 99.4 | 99.4 | 99.4 | 99.2 | 97.1 | 89.9 | 91.0 | 98.9 | 96.6 | 97.4 | 86.0 | 85.1 | 51.3 |
|  | *CHS2* | 94.8 |  | 99.4 | 99.4 | 99.4 | 99.2 | 99.2 | 99.4 | 96.9 | 90.2 | 91.2 | 98.7 | 96.3 | 97.1 | 86.3 | 84.9 | 51.3 |
|  | *CHS3a* | 98.4 | 95.2 |  | 100.0 | 100.0 | 99.4 | 99.4 | 99.4 | 97.1 | 89.9 | 91.0 | 98.9 | 96.6 | 97.4 | 86.3 | 85.1 | 51.3 |
|  | *CHS3b* | 98.5 | 95.1 | 99.9 |  | 100.0 | 97.8 | 99.4 | 97.9 | 92.3 | 81.4 | 68.8 | 97.6 | 91.1 | 92.6 | 85.6 | 72.9 | 51.3 |
|  | *CHS3c* | 98.5 | 95.1 | 99.9 | 100.0 |  | 97.8 | 99.4 | 97.9 | 92.3 | 81.4 | 68.8 | 97.6 | 91.1 | 92.6 | 85.6 | 72.9 | 56.4 |
|  | *CHS4a* | 98.2 | 95.1 | 97.7 | 99.4 | 99.4 |  | 100.0 | 99.7 | 97.1 | 89.7 | 90.7 | 98.9 | 96.6 | 97.4 | 86.5 | 85.1 | 51.3 |
|  | *CHS4b* | 98.2 | 95.1 | 97.7 | 97.8 | 97.8 | 100.0 |  | 99.7 | 92.6 | 80.4 | 68.2 | 97.5 | 91.2 | 92.8 | 87.1 | 72.8 | 56.0 |
|  | *CHS5* | 98.0 | 95.3 | 97.8 | 99.4 | 99.4 | 99.7 | 99.7 |  | 96.9 | 89.9 | 91.0 | 98.7 | 96.3 | 97.1 | 86.8 | 85.1 | 51.3 |
|  | *CHS6* | 92.8 | 92.9 | 92.4 | 97.1 | 97.1 | 92.6 | 97.1 | 92.5 |  | 89.7 | 90.2 | 96.6 | 97.4 | 99.2 | 83.7 | 83.8 | 52.4 |
|  | *CHS7* | 81.3 | 81.0 | 81.4 | 89.9 | 89.9 | 80.4 | 89.7 | 80.6 | 80.2 |  | 98.7 | 89.2 | 89.4 | 89.9 | 77.3 | 82.8 | 51.9 |
|  | *CHS8* | 69.0 | 68.4 | 68.8 | 91.0 | 91.0 | 68.2 | 90.7 | 68.2 | 67.7 | 82.0 |  | 90.2 | 89.9 | 90.4 | 78.4 | 83.1 | 51.9 |
|  | *CHS9* | 97.3 | 95.2 | 97.5 | 98.9 | 98.9 | 97.5 | 98.9 | 97.6 | 92.8 | 81.0 | 68.6 |  | 96.1 | 96.9 | 85.8 | 84.6 | 50.8 |
|  | *CHS10* | 91.4 | 91.8 | 91.2 | 96.6 | 96.6 | 91.2 | 96.6 | 91.1 | 95.2 | 80.3 | 67.9 | 91.5 |  | 97.6 | 83.5 | 83.6 | 51.9 |
|  | *CHS11* | 93.1 | 93.2 | 92.7 | 97.4 | 97.4 | 92.8 | 97.4 | 92.7 | 99.5 | 80.3 | 67.7 | 93.0 | 95.4 |  | 84.0 | 84.3 | 52.4 |
|  | *CHS12* | 85.5 | 83.2 | 85.6 | 86.3 | 86.3 | 87.1 | 86.5 | 87.4 | 80.5 | 70.2 | 58.0 | 85.0 | 79.4 | 80.7 |  | 73.4 | 43.0 |
|  | *CHS13* | 73.0 | 73.2 | 72.9 | 85.1 | 85.1 | 72.8 | 85.1 | 72.8 | 72.1 | 70.8 | 59.6 | 73.3 | 70.7 | 72.2 | 63.1 |  | 52.9 |
|  | *CHS14* | 56.3 | 55.7 | 56.5 | 56.4 | 51.3 | 56.0 | 51.3 | 56.0 | 56.5 | 56.5 | 50.9 | 56.0 | 56.2 | 56.3 | 47.5 | 57.4 |  |
