# Supplementary material for: Genome-wide identification and localization of chalcone synthase family in soybean (Glycine max [L]Merr)
Source: BMC Plant Biol. 2018 Dec 4;18:325. doi: 10.1186/s12870-018-1569-x (PMC6278125; doi:10.1186/s12870-018-1569-x)
Supplement: Supplementary file 2 — Promoter sequence identity matrix of GmCHS genes. (DOCX 20 kb) [file 12870_2018_1569_MOESM2_ESM.docx]

Table S2. Promoter sequence (1000 bp) identity matrix of *GmCHS*s

|  | **Promoter sequence (% identity)** | | | | | | | | | | | | | | | | |
| --- | --- | --- | --- | --- | --- | --- | --- | --- | --- | --- | --- | --- | --- | --- | --- | --- | --- |
|  | ***CHS1*** | ***CHS2*** | ***CHS3a*** | ***CHS3b*** | ***CHS3c*** | ***CHS4a*** | ***CHS4b*** | ***CHS5*** | ***CHS6*** | ***CHS7*** | ***CHS8*** | ***CHS9*** | ***CHS10*** | ***CHS11*** | ***CHS12*** | ***CHS13*** | ***CHS14*** |
| ***CHS1*** |  | 35.8 | 35.0 | 0.6 | 35.7 | 37.0 | 34.8 | 41.4 | 38.8 | 31.3 | 33.9 | 38.6 | 38.4 | 37.2 | 41.4 | 34.0 | 33.3 |
| ***CHS2*** |  |  | 39.3 | 2.4 | 41.2 | 40.6 | 37.6 | 38.9 | 39.3 | 31.0 | 31.8 | 37.1 | 33.9 | 38.4 | 38.9 | 32.6 | 31.9 |
| ***CHS3a*** |  |  |  | 2.6 | 48.9 | 38.4 | 35.2 | 35.0 | 37.4 | 29.8 | 30.4 | 35.2 | 33.3 | 35.8 | 35.0 | 29.6 | 30. |
| ***CHS3b*** |  |  |  |  | 2.6 | 1.6 | 11.0 | 2.0 | 1.7 | 1.2 | 1.9 | 1.6 | 2.0 | 1.8 | 2.0 | 1.2 | 0.4 |
| ***CHS3c*** |  |  |  |  |  | 39.8 | 37.6 | 37.3 | 39.6 | 33.1 | 34.2 | 37.5 | 35.3 | 38.9 | 37.3 | 31.6 | 32.0 |
| ***CHS4a*** |  |  |  |  |  |  | 81.6 | 40.0 | 48.2 | 32.8 | 32.7 | 39.9 | 37.8 | 47.8 | 40.0 | 30.3 | 31.8 |
| ***CHS4b*** |  |  |  |  |  |  |  | 37.8 | 43.7 | 30.4 | 30.8 | 36.3 | 34.1 | 41.4 | 37.8 | 27.7 | 29.8 |
| ***CHS5*** |  |  |  |  |  |  |  |  | 40.9 | 34.1 | 36.7 | 45.8 | 40.7 | 40.3 | 100.0 | 33.2 | 30.5 |
| ***CHS6*** |  |  |  |  |  |  |  |  |  | 32.4 | 34.2 | 41.8 | 45.8 | 92.5 | 40.9 | 32.1 | 33.0 |
| ***CHS7*** |  |  |  |  |  |  |  |  |  |  | 44.0 | 32.8 | 29.3 | 30.9 | 34.1 | 31.9 | 30.8 |
| ***CHS8*** |  |  |  |  |  |  |  |  |  |  |  | 34.1 | 31.6 | 33.6 | 36.7 | 32.6 | 28.4 |
| ***CHS9*** |  |  |  |  |  |  |  |  |  |  |  |  | 51.7 | 40.8 | 45.8 | 32.6 | 32.0 |
| ***CHS10*** |  |  |  |  |  |  |  |  |  |  |  |  |  | 45.8 | 40.7 | 32.9 | 29.8 |
| ***CHS11*** |  |  |  |  |  |  |  |  |  |  |  |  |  |  | 40.3 | 30.6 | 31.7 |
| ***CHS12*** |  |  |  |  |  |  |  |  |  |  |  |  |  |  |  | 33.2 | 30.5 |
| ***CHS13*** |  |  |  |  |  |  |  |  |  |  |  |  |  |  |  |  | 31.1 |
| ***CHS14*** |  |  |  |  |  |  |  |  |  |  |  |  |  |  |  |  |  |
