# Supplementary material for: Genome-wide identification and localization of chalcone synthase family in soybean (Glycine max [L]Merr)
Source: BMC Plant Biol. 2018 Dec 4;18:325. doi: 10.1186/s12870-018-1569-x (PMC6278125; doi:10.1186/s12870-018-1569-x)
Supplement: Supplementary file 4 — List of genes within 134.56 kb region containing GmCHS on chromosome 8 in soybean. (DOCX 19 kb) [file 12870_2018_1569_MOESM4_ESM.docx]

Table S4. List of genes within 134.56 kb region containing *GmCHS* on chromosome 8 in soybean.

| Locus ID | Chromosome location | | Strand | | Annotation | |
| --- | --- | --- | --- | --- | --- | --- |
| Glyma.08G109200 | | 8384742-8386542 | | + | | Chalcone synthase (*GmCHS5)* |
| Glyma.08G109300 | | 8387509-8391327 | | - | | Chalcone synthase (*GmCHS3a)* |
| Glyma.08G109400 | | 8391364-8394840 | | + | | Chalcone synthase (*GmCHS1*) |
| Glyma.08G109500 | | 8397944-8399751 | | + | | Chalcone synthase (*GmCHS9)* |
| Glyma.08G109600 | | 8404346-8411383 | | + | | Filamin/ABP280 repeat, RRM, splicing factor |
| Glyma.08G109700 | | 8419650-8422508 | | + | | DNAJ domain |
| Glyma.08G109800 | | 8423696-8424408 | | - | | Not functionally annotated |
| Glyma.08G109900 | | 8431287-8436291 | | + | | Cytochrome P450; Premnaspirodiene oxygenase |
| Glyma.08G110000 | | 8438346-8442806 | | - | | Transferase family |
| Glyma.08G110100 | | 8456916-8466199 | | - | | Not functionally annotated |
| Glyma.08G110200 | | 8471393-8473156 | | + | | MYB-like DNA binding domain |
| Glyma.08G110300 | | 8475793-8477410 | | + | | Chalcone synthase (*GmCHS3c)* |
| Glyma.08G110400 | | 8478834-8480215 | | - | | Chalcone synthase (*GmCHS12)* |
| Glyma.08G110500 | | 8504479-8506020 | | - | | Chalcone synthase (*GmCHS4b)* |
| Glyma.08G110600 | | 8507912-8510245 | | - | | Not functionally annotated |
| Glyma.08G110700 | | 8513952-8515719 | | + | | Chalcone synthase (*GmCHS4a)* |
| Glyma.08G110800 | | 8516752-8516997 | | - | | Not functionally annotated |
| Glyma.08G110900 | | 8517799-8519303 | | - | | Chalcone synthase (*GmCHS3b)* |

+ and – indicate sense and antisense strand, respectively.
