# Supplementary material for: Genome-wide identification and localization of chalcone synthase family in soybean (Glycine max [L]Merr)
Source: BMC Plant Biol. 2018 Dec 4;18:325. doi: 10.1186/s12870-018-1569-x (PMC6278125; doi:10.1186/s12870-018-1569-x)
Supplement: Supplementary file 6 — Sequences of oligonucleotides used in the study. (DOCX 24 kb) [file 12870_2018_1569_MOESM6_ESM.docx]

**Table S6** Sequences of oligonucleotides used in the study.

| **Gene** | **Primer Name** | **Sequence (5’ to 3’)** | **Amplicon size (bp)** | **Use** |
| --- | --- | --- | --- | --- |
| *GmCHS1* | CHS1-F | GCCACGAGTCAGAGGAAGCA | 1343 | Nested PCR |
|  | CHS1-R | GAGAGTGACACTGCGGAGCA |  |  |
| *GmCHS2* | CHS2-F | CACTCCGCTCCTCCTCTCAC | 1279 |  |
|  | CHS2-R | GACACTGACACTGCGGAGCA |  |  |
| *GmCHS3* | CHS10-F | ATCCAACCTTCCAAACCAGT | 1327 |  |
|  | CHS10-R | GACAGTGACACTGCGTAGCA |  |  |
| *GmCHS4* | CHS4-F1 | CGTAGATAGCTACTACTTCACTTTCA | 1220 |  |
|  | CHS4/5/12-R | GGGGACCACTTTGTACAAGAAAGCTGGGTCGACAGTGACACTGCGGAGTA |  |  |
| *GmCHS5* | CHS12-F | CGCATCTACCTACCCACCCT | 1252 |  |
|  | CHS4/5/12-R | GGGGACCACTTTGTACAAGAAAGCTGGGTCGACAGTGACACTGCGGAGTA-3’ |  |  |
| *GmCHS6* | CHS6-F | GGTTCAAAGAAGCTTAGTTGTTGGT | 1209 |  |
|  | CHS6-R | GACTGTGACACTGCGAAGCA |  |  |
| *GmCHS9* | CHS9-F | CGGGGAACCTACCCACCCTT | 1342 |  |
|  | CHS3/9-R | GGGGACCACTTTGTACAAGAAAGCTGGGTcGACAGTGACACTGCGGAGCA |  |  |
| *GmCHS10* | CHS10-F | ATCCAACCTTCCAAACCAGT | 1327 |  |
|  | CHS10-R | GACAGTGACACTGCGTAGCA |  |  |
| *GmCHS11* | CHS11-F | TCCCCATCATTCATATCATAGCCT | 1274 |  |
|  | CHS6/11-R | GGGGACCACTTTGTACAAGAAAGCTGGGTcGACTGTGACACTGCGAAGCA |  |  |
| *GmCHS1* | CHS1-F1 | GGGGACAAGTTTGTACAAAAAAGCAGGCTTCTGCTACTTCCCACTTCCATTCTTTTCT | 1203 | Subcellular localization |
|  | CHS1-R1 | GGGGACCACTTTGTACAAGAAAGCTGGGTCGAGAGTGACACTGCGGAGCA |  |  |
| *GmCHS2* | CHS2/5/12-F1 | GGGGACAAGTTTGTACAAAAAAGCAGGCTTCATGGTGAGTGTTGAAGAGATCCGTC | 1164 |  |
|  | CHS2-R1 | GGGGACCACTTTGTACAAGAAAGCTGGGTCGACACTGACACTGCGGAGCA |  |  |
| *GmCHS3* | CHS4/6/10/11-F1 | GGGGACAAGTTTGTACAAAAAAGCAGGCTTCATGGTGAGTGTTGAAGAGATTCGTAA | 1164 |  |
|  | CHS10-R1 | GGGGACCACTTTGTACAAGAAAGCTGGGTCGACAGTGACACTGCGTAGCA |  |  |
| *GmCHS4* | CHS4/6/10/11-F1 | GGGGACAAGTTTGTACAAAAAAGCAGGCTTCATGGTGAGTGTTGAAGAGATTCGTAA | 1164 |  |
|  | CHS4/5/12-R | GGGGACCACTTTGTACAAGAAAGCTGGGTCGACAGTGACACTGCGGAGTA |  |  |
| *GmCHS5* | CHS2/5/12-F1 | GGGGACAAGTTTGTACAAAAAAGCAGGCTTCATGGTGAGTGTTGAAGAGATCCGTC | 1164 |  |
|  | CHS4/5/12-R | GGGGACCACTTTGTACAAGAAAGCTGGGTCGACAGTGACACTGCGGAGTA |  |  |
| *GmCHS6* | CHS4/6/10/11-F1 | GGGGACAAGTTTGTACAAAAAAGCAGGCTTCATGGTGAGTGTTGAAGAGATTCGTAA | 1164 |  |
|  | CHS6/11-R | GGGGACCACTTTGTACAAGAAAGCTGGGTcGACTGTGACACTGCGAAGCA |  |  |
| *GmCHS7* | CHS7-F | GGGGACAAGTTTGTACAAAAAAGCAGGCTTCATGGTTAGCGTAGCTGAGATCAGGCAGGC | 1167 |  |
|  | CHS7R | GGGGACCACTTTGTACAAGAAAGCTGGGTCGATGGCCACACTATGCAAAACAACAGTTTC |  |  |
| *GmCHS8* | CHS8-F | GGGGACAAGTTTGTACAAAAAAGCAGGCTTCATGGTGAGCGTAGCTGAGATCCGCC | 1167 |  |
|  | CHS8R | GGGGACCACTTTGTACAAGAAAGCTGGGTCGATGGCCACACTGCGCAGAACAACAGTTTC |  |  |
| *GmCHS9* | CHS9-F1 | GGGGACAAGTTTGTACAAAAAAGCAGGCTtcATGGTGAGTGTTGAAGCAATCC | 1164 |  |
|  | CHS3/9-R | GGGGACCACTTTGTACAAGAAAGCTGGGTcGACAGTGACACTGCGGAGCA |  |  |
| *GmCHS10* | CHS4/6/10/11-F1 | GGGGACAAGTTTGTACAAAAAAGCAGGCTTCATGGTGAGTGTTGAAGAGATTCGTAA | 1164 |  |
|  | CHS10-R1 | GGGGACCACTTTGTACAAGAAAGCTGGGTCGACAGTGACACTGCGTAGCA-3’ |  |  |
| *GmCHS11* | CHS4/6/10/11-F1 | GGGGACAAGTTTGTACAAAAAAGCAGGCTTCATGGTGAGTGTTGAAGAGATTCGTAA | 1164 |  |
|  | CHS6/11-R | GGGGACCACTTTGTACAAGAAAGCTGGGTcGACTGTGACACTGCGAAGCA |  |  |
| *GmCHS13* | CHS13-F | GGGGACAAGTTTGTACAAAAAAGCAGGCTTCTTCTTCCCCCTCCACCCACC | 1327 |  |
|  | CHS13-R | GGGGACCACTTTGTACAAGAAAGCTGGGTCTCCCTCCAAGGGAACACTGT |  |  |
| *GmCHS14* | GmCHS4-F6 | GGGGACAAGTTTGTACAAAAAAGCAGGCTTCATGGAGAGGGAACAAATTGGAGGA | 1164 |  |
|  | GmCHS14-R | GGGGACCACTTTGTACAAGAAAGCTGGGTCACATGGATAACTACGTAAAAGCACAG |  |  |
| *mcherry* | AvrII-mCherry-F | AAACCTAGGGTGAGCAAGGGCGAGGAGGA | 737 | mcherry  vector |
|  | XbaI-6His-mCherry-R | AAATCTAGATCCATGATGATGATGATGATGCTTGTACAGCTCGTCCATG |  |  |
| *GmCHS9* | qCHS9-F | CACGCGTCTACCTTAACGGG | 165 | qPCR |
|  | qCHS9-R | GCTAGCTTGCACCAAAGAATGA |  |  |
| *GmCHS10* | qCHS10-F | TTCCTTCTCTTTTGCTTCATTTTGT | 155 |  |
|  | qCHS10-R | AGTGAGCCACAAAGAATGTTTTA |  |  |
| *GmCHS11* | qCHS11-F | TGCAAAGTGAATAGCTTTTGTCCA | 100 |  |
|  | qCHS11-R | CCTTGTTCACAATTTCACAAAGTCC |  |  |
| *GmCHS13* | qCHS13-F | CCCTCGGAAATACCAGGGAA | 169 |  |
|  | qCHS13-R | CGAAAACGAAATGCAATGCCG |  |  |
| *GmCHS14* | qCHS14-F | TTCCGGGTCACAAATAGCGA | 176 |  |
|  | qCHS14-R | GTGGGGACATTCTGTCGTGT |  |  |
